# Differential Valuation and Learning From Social and Nonsocial Cues in Borderline Personality Disorder

**DOI:** 10.1016/j.biopsych.2018.05.020

**Published:** 2018-12-01

**Authors:** Sarah K. Fineberg, Jacob Leavitt, Dylan S. Stahl, Sharif Kronemer, Christopher D. Landry, Aaron Alexander-Bloch, Laurence T. Hunt, Philip R. Corlett

**Affiliations:** a Department of Psychiatry, Yale University, New Haven, Connecticut; b Yale Child Study Center, Yale University, New Haven, Connecticut; c Department of Neurology, Yale University, New Haven, Connecticut; d Department of Psychology, University of Houston, Houston, Texas; e Columbia University College of Physicians and Surgeons, Columbia University, New York, New York; f Wellcome Centre for Integrative Neuroimaging, University of Oxford, Oxford, United Kingdom; g Oxford Health National Health Service Foundation Trust, Oxford, United Kingdom

**Keywords:** Associative learning, Borderline personality disorder, Computational psychiatry, Prediction error, Social cognition, Trust

## Abstract

**Background:**

Volatile interpersonal relationships are a core feature of borderline personality disorder (BPD) and lead to devastating disruption of patients’ personal and professional lives. Quantitative models of social decision making and learning hold promise for defining the underlying mechanisms of this problem. In this study, we tested BPD and control subject weighting of social versus nonsocial information and their learning about choices under stable and volatile conditions. We compared behavior using quantitative models.

**Methods:**

Subjects (*n* = 20 BPD, *n* = 23 control subjects) played an extended reward learning task with a partner (confederate) that requires learning about nonsocial and social cue reward probability (the social valuation task). Task experience was measured using language metrics: explicit emotions/beliefs, talk about the confederate, and implicit distress (using the previously established marker self-referentiality). Subjects’ weighting of social and nonsocial cues was tested in mixed-effect regression models. Subjects’ learning rates under stable and volatile conditions were modeled (Rescorla–Wagner approach) and group × condition interactions tested.

**Results:**

Compared to control subjects, BPD subject debriefings included more mentions of the confederate and less distress language. BPD subjects also weighted social cues more heavily but had blunted learning responses to (nonsocial and social) volatility.

**Conclusions:**

This is the first report of patient behavior in the social valuation task. The results suggest that BPD subjects expect higher volatility than control subjects. These findings lay the groundwork for a neurocomputational dissection of social and nonsocial belief updating in BPD, which holds promise for the development of novel clinical interventions that more directly target pathophysiology.

Learning whom to trust and when to revise trust attributions is a difficult but important task. People exhibiting extremes in trust can experience significant distress and personal risk, as in the very low trust that characterizes paranoia 1, 2 and the very high trust in Williams syndrome (3) or amygdalar lesions (4). In borderline personality disorder (BPD), trust is unstable, and interpersonal relationships involve recurrent episodes of rupture and repair. People with BPD suffer immensely and attempt suicide at 50-fold the rate of the general population (5). Research investigating the mechanism of interpersonal problems in BPD is needed to identify targets for rational and effective treatment innovation. Low initial trust and rupture-promoting behavior in BPD have been modeled in the 10-round trust game, a brief economic exchange task with a partner (6). We aimed to extend those data by examining responses of people with BPD to instability of social and nonsocial information.

For this study, we used the social valuation task (SVT), a laboratory-based reinforcement learning paradigm with social and nonsocial dimensions (7). The nonsocial dimensions are the color and the number on cards from which the subject chooses for a potential points reward. The social dimension is advice from a confederate. Based on carefully computed contingencies, we can independently assess weighting of and learning rates for the social and nonsocial dimensions. Healthy control subjects use both nonsocial and social dimensions (7), and they learn faster about each dimension when it is less reliable (8). Functional magnetic resonance imaging dissociated social and nonsocial learning signals regionally (7).

Weighting of social versus nonsocial cues in the SVT in community samples correlates with self-reported traits. In healthy adults, self-reported autistic traits were directly correlated with poorer overall task performance and inversely correlated with weighting social over nonsocial cues (9). Also, subjects with more autistic traits were worse at avoiding the influence of bad advice during the “volatile phase” of the task, when reward for the social cue was most unreliable. In another study, healthy women reported degree of psychopathic traits (10). As with autism score, the psychopathy subscale “social potency”—a measure of the ability to charm and manipulate others—was inversely correlated with weighting of social cues, whereas “fearlessness” was inversely correlated with use of the nonsocial cue, and “stress immunity” was inversely correlated with weighting of both cue types. Also, Diaconescu *et al.* (11) reported that in healthy men, self-reported stable social attributions correlated with stable beliefs about their game partner in a deception-free two-player version of the SVT. In sum, weighting of cues correlated with traits as expected. Autistic and manipulative traits correlated with decreased ability to make use of incoming social data, and stable social beliefs in self-reports correlated with stable social beliefs in the game.

We report here the first test of SVT behavior in a patient population. We model both weighting of social versus nonsocial cues and learning rates in response to changes in social and nonsocial reward volatility. Our main hypotheses about behavioral differences between BPD and control subjects were formulated in light of others’ work about BPD social experience, including interpersonal hypersensitivity, rejection sensitivity, and hypermentalization (12). We expected that people with BPD would be highly sensitive to small changes in the social environment (H1–H3) and would change their behavior quickly in response to instability of the social environment (H4 and H5).

H1: In BPD, social cues would be weighted more heavily than nonsocial cues.
H2: Social cues would be weighted more heavily by BPD than control subjects.
H3: Negative social cues would be weighted more heavily, and positive social cues would be weighted less heavily, by BPD than control subjects.
H4: Learning rate would increase more in during the volatile period in BPD than in control subjects.
H5: In BPD, learning rate would increase more in response to social volatility than nonsocial volatility.

We complemented our quantitative models of subject decisions with analysis of subjects’ experience. Consistent with our expectation that people with BPD would be more socially focused and responsive, especially to negative social data, we hypothesized that BPD subjects would express more surprise, suspicion, distress, and focus on the confederate. We expected that people (and BPD > control subjects) would experience implicit distress owing to the periods of volatile and untrustworthy advice in the task, or owing to hearing at the end that the confederate was not in fact another player, and that we had intentionally misled them. We measured implicit distress by counting self-referential language (words like I, me, and mine), as they are known to increase with distress in mental and physical illnesses 13, 14, 15.

## Methods and Materials

### Ethics

This protocol was written and conducted in accordance with the Declaration of Helsinki and was approved by the Yale Institutional Review Board (protocol 1211011104).

### Subjects

Women 18 to 65 years of age were recruited from the community, and subjects were identified who met criteria for either the healthy control or BPD group (Tables 1 and 2) (see Supplemental Methods and Materials for details).

**Table 1:** Subject Demographics

	Control Subjects	BPD Subjects
*n*	23	20
Age, Years, Mean ± SEM (Range)	33.86 ± 2.93 (18–60)	35.80 ± 2.91 (18–63)
Education, Years, Mean ± SEM (Range)	14.78 ± 0.57 (10–19)	14.20 ± 0.54 (11–20)
Ethnicity, %		
Asian	13	10
Black	30	15
Hispanic	9	5
White	44	55
Not reported	4	15
Taking Psychiatric Medications, %	0	45^a^
Antidepressant	0	15
Mood stabilizer	0	25
Antipsychotic	0	15
Benzodiazepine	0	10
Current Relationship, %		
None	30	35
In a relationship	26	35
Married	13	10
No answer	31	20
Has Children, %		
Yes	22	17
No	48	55
No answer	30	28
Current Work, %		
None	26	45
0–20 hours/week	13	15
≥20 hours/week	22	15
In school	30	10
No answer	9	15

BPD, borderline personality disorder.

a Note that some individuals in the BPD group were taking multiple psychiatric medications.

**Table 2:** Subject Characteristics

	Control Subjects	BPD Subjects	*p* Value
NAART	20.90 ± 2.09	19.20 ± 1.69	.53
SCID2-BPD	0.90 ± 0.28	9.00 ± 0.76	<.001
BSL-23	5.95 ± 1.60	33.00 ± 4.29	<.001
BDI	3.10 ± 1.06	21.40 ± 2.95	<.001
BAI	7.75 ± 2.32	23.00 ± 2.86	<.001

Mean and SEM scores are displayed for the NAART (reading test), two different BPD self-reports (SCID2-BPD and BSL-23), depression self-report (BDI), and anxiety self-report (BAI). *t* tests comparing control with BPD subject scores revealed no differences in reading score between groups but significant differences in each of the self-reports.

BAI, Beck Anxiety Inventory; BDI, Beck Depression Inventory; BPD, borderline personality disorder; BSL-23, Borderline Symptom List 23; NAART, North American Adult Reading Test; SCID2-BPD, Structured Clinical Interview for DSM-IV Axis II Disorders–Borderline Personality Disorder.

### Self-report Scales

Refer to the Supplemental Methods and Materials for details regarding self-report scales used in the study.

### Social Valuation Task Design

**Figure 1:**
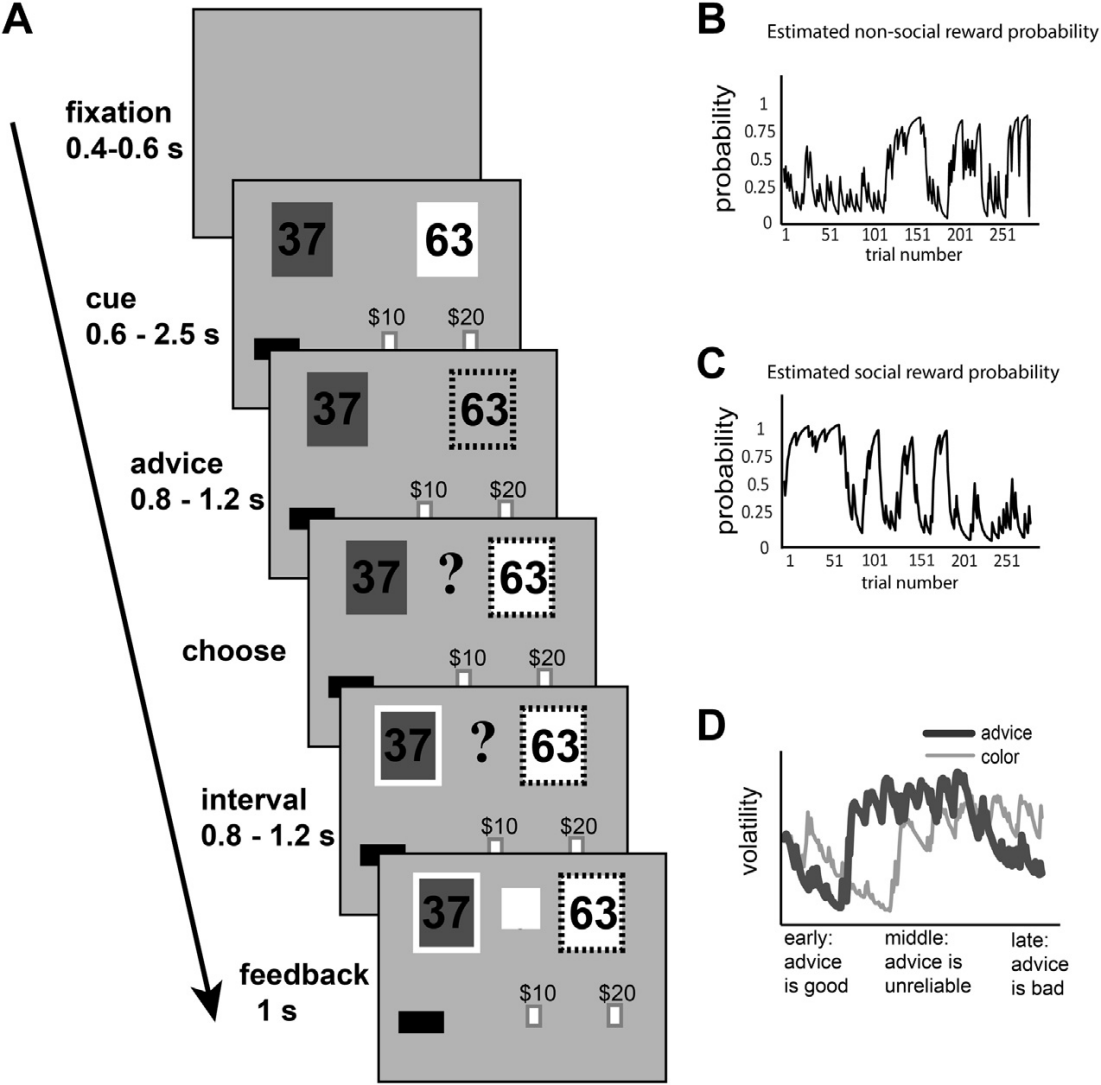
Task design. **(A)** Cartoon of task phases including elements displayed to the subject in each phase and timing of the phases. Note that the subject makes her choice in the “choose” phase and sees the correct answer in the “feedback” phase. These six phases are repeated in each of 290 trials. **(B)** Changing probability of reward for the nonsocial cue (green) over time. **(C)** Changing probability of reward for the social cue (advice) over time—note the separate pattern from the nonsocial cue. **(D)** Changes in the volatility of the nonsocial (thin line) and social (thick line) reward probabilities represented as volatility over time. The x-axis is again time (trial number), and quality of advice is indicated as it changes from low-volatility and trustworthy advice at the beginning through a period of high-volatility advice, to a period of low-volatility but untrustworthy advice at the end of the task.

The SVT was implemented as described by Behrens *et al.* (7) (Figure 1). For a detailed description of the task, refer to the Supplemental Methods and Materials.

### Confederate

The task confederates were 20- to 30-year-old white women trained for consistent interaction with subjects and consistent performance during the demonstration task.

### Debriefing

Immediately after the task, subjects were audiorecorded talking with study staff in response to a list of specific questions and statements about the task experience. We asked four questions before the disclosure that the confederate was not actually a second game player, then two more questions after the disclosure. We examined the transcribed language from the debriefings. We counted the number of times that the subject mentioned the confederate.

To capture shifts in emotional state before versus after the disclosure, we examined the use of self-referential pronouns in subject speech (14). Transcribed speech was analyzed with Linguistic Inquiry and Word Count (16), which returns the frequency of specific categories (we used “first-person pronouns”) as count/total words. We used repeated measures analysis of variance to test for interaction between time (before vs. after disclosure) and group (BPD vs. control).

### Modeling Task Behavior: Relative Cue Weighting

Variables influencing subject decisions were examined with mixed models in the statistical program R using the package lme4 (17). Probability and volatility values were those derived by Behrens *et al.* (8) from their Bayes optimal model. Nonsocial variables were points (difference between point magnitude for green and point magnitude for blue), probability of green's being correct, and volatility of green’s being correct. Social variables were current trial advice, current advice weighted by probability that advice is correct, current advice weighted by volatility of advice being correct, and refusing current advice after recent betrayal. We also tested a series of time windows on recent betrayal (incorrect advice) or help (correct advice): within x trials, where x = 1, 3, 4, 5, 6, or 7. Each variable was centered and *Z*-scored to facilitate comparison of coefficients across factors. The impact of clinical group was tested separately for each of the above predictor variables *v* (modeled as fixed effects in the mixed models). Likelihood ratio tests were used to compare nested models. For details of model comparisons, refer to the Supplemental Methods and Materials.

### Modeling Task Behavior: Learning Rates

Subject learning rates were modeled using the R package hBayesDM and the function bandit2arm_delta using default parameters (18). This package calculates mean learning rates for each subject based on the Rescorla–Wagner (delta) equation (see details in the Supplemental Methods and Materials).

The SVT has two phases for the nonsocial cue: stable (trials 1–130) and volatile (trials 131–290) (Figure 1B, D). There are three phases for the social cue: stable reliable (trials 1–70), volatile (trials 71–210), and stable unreliable (trials 211–290) (Figure 1C, D). In our analyses, learning for each cue was modeled based on the trials in each phase. Repeated measures analysis of variance was used to test for group × phase interaction for each cue.

## Results

### Demographics

**Figure 2:**
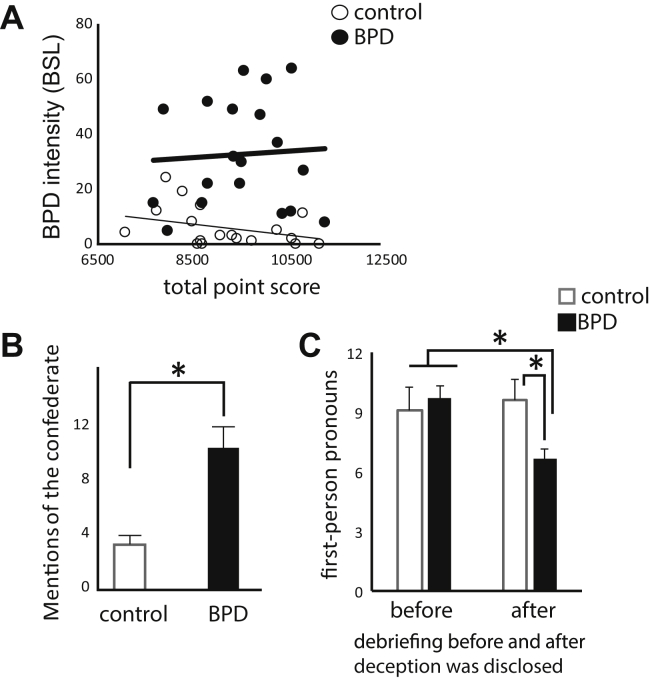
Task experience. **(A)** Borderline personality disorder (BPD) and control subjects achieve similar final point scores in the game (*t* test, *p* = .345), and the final point score does not correlate with BPD symptom score in either group (Pearson correlation, control *r* = −.352, *p* = .140; BPD *r* = .061, *p* = .804). **(B)** In the posttask debriefing, BPD subjects talk significantly more about the confederate than do control subjects. Error bars represent SEM (*t* test, *p* = .01). **(C)** In the posttask debriefing, BPD and control subjects refer to themselves with similar frequency before the deception is revealed. However, after they hear that the computer, not the confederate, was providing the advice, self-referential language is significantly less in BPD than in control subjects. In a repeated measures analysis of variance, there was a trend level difference by time (*F* = 3.03, *p* = .09) and a significant difference for the time × group interaction (*F* = 6.16, *p* = .02). BSL, Borderline Symptom List.

Subjects (control *n* = 23, BPD *n* = 20) were matched on age (*t*_39_ = –0.47, *p* = .641) and education (for years in school *t*_39_ = 0.751, *p* = .457; for reading score *t*_39_ = 0.631, *p* = .53) (Table 1). BPD subjects had significantly more severe BPD, depression, and anxiety (Table 2). All the subjects were able to complete the task, and their final point scores did not differ by group or symptom burden (Figure 2A).

### BPD Patients Talk More About the Confederate, But Show Lower Implicit Distress in Response to Task

As a preliminary test for enhanced focus on social cues in BPD, we counted references to the confederate in audio recordings of the post task debriefing. There were more mentions of the confederate in BPD versus control subject language (Figure 2B) (mean BPD = 11.60, SE = 2.34, mean control = 4.77, SE = 1.34; *t*_21_ = −2.67, *p* = .01). The two groups did not differ in expressed surprise (mean BPD = 0.8, SE = 0.29; mean control = 0.77, SE = 0.23; *t*_21_ = −0.08, *p* = .93), distress (mean BPD = 0, SE = 0; mean control = 0.15, SE = 0.10, *t*_21_ = 1.3, *p* = .21), or suspicion (mean BPD = 0.10, SE = 0.10; mean control = 0.08, SE = 0.08, *t*_21_ = –0.19, *p* = .85).

Though none of the subjects demonstrated overt distress during or after the task, we also tested for implicit distress. We used a previously established language measure: frequency of self-referential words (see introduction). We analyzed subject language before and after we revealed the deception (that the social cues were controlled by the computer, not the human confederate). Control subjects used similar levels of self-referential words before and after disclosure. BPD subjects used similar levels to control subjects before disclosure, but significantly fewer afterward (time × group interaction *F* = 6.16, *p* = .02) (Figure 2C). This suggests that in BPD subjects, distress decreased after the deception was revealed.

### H1/2: People With BPD Weighted Social More Heavily Than Nonsocial Cues

**Figure 3:**
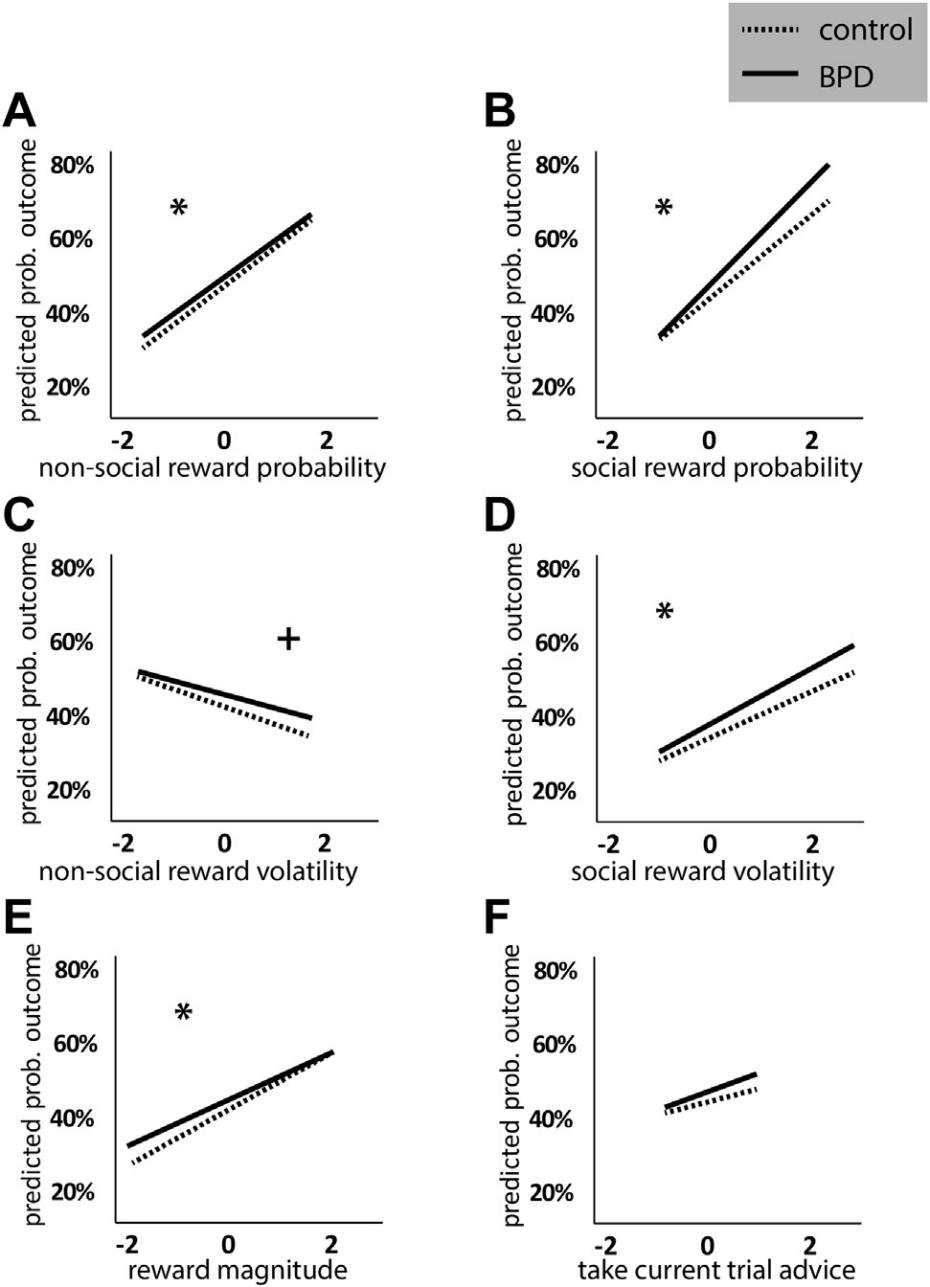
Borderline personality disorder (BPD) and control subjects use cues differently to make decisions. Mixed-effect logistic regression models tested the effects of nonsocial and social predictors on subject choices. The predicted probability of choosing green is plotted over *Z*-scored values of each predictor. Both nonsocial predictors **(A, C, E)** and social predictors **(B, D)** performed differently between groups [except **(F)**, current trial advice]. Significant χ^2^ for models with vs. without group term at *p* < .05 is indicated by an asterisk (*). Trending χ^2^ for models with vs. without group term at *p* < .1 is indicated by a plus symbol (+).

We tested the impact of nonsocial and social cues on subject choices in the SVT (Figure 3). To test our first and second hypotheses, we examined the weights of nonsocial and social cues in subject decisions. Each of the variables was a significant contributor to subject decisions and contributed differently to decisions between groups. Specifically, BPD participants were more likely than control subjects to choose green when the reward probability was higher (likelihood ratio χ^2^ statistic = −4.03, *p* = .045, reward probability coefficient = 0.41, group coefficient = 0.11) (Figure 3A) and less likely than control subjects to choose green when the likelihood of reward became more volatile (likelihood ratio χ^2^ statistic = −3.48, trend level significance *p* = .062, reward volatility coefficient = −0.17, group coefficient = 0.10) (Figure 3C). They were also more likely than control subjects to choose green if the difference between points for green and points for blue was larger (likelihood ratio χ^2^ statistic = −4.07, *p* = .044, Δ points coefficient = 0.30, group coefficient = 0.11) (Figure 3E). BPD participants were more likely to go with the advice if the reward probability was higher compared with control subjects (likelihood ratio χ^2^ statistic = −5.98, *p* = .015, social reward probability coefficient = 0.46, group coefficient = 0.12) (Figure 3B).

Of interest, and perhaps surprising, is that both groups (BPD > control subjects) were also more likely to take the advice when social reward likelihood was more volatile (likelihood ratio χ^2^ statistic = −4.96, *p* = .026, social volatility coefficient = 0.21, group coefficient = 0.12) (Figure 3D). However, the model describing outcomes predicted by group and current trial advice [what Behrens *et al.* (7) termed “blindly following advice”] did not detect statistical differences (Figure 3F). In sum, we found that people with BPD made significant use of both nonsocial and social cues. Interestingly, people with BPD weighted both social and nonsocial cues more heavily than control subjects, although between-group differences were larger for weighting of social reward probability than for nonsocial reward probability (based on magnitude of difference between regression lines; as noted, χ^2^ tests were significant in both cases).

### H3: People With BPD Used Positive Social Cues More Than Negative Social Cues to Make Decisions

**Figure 4:**
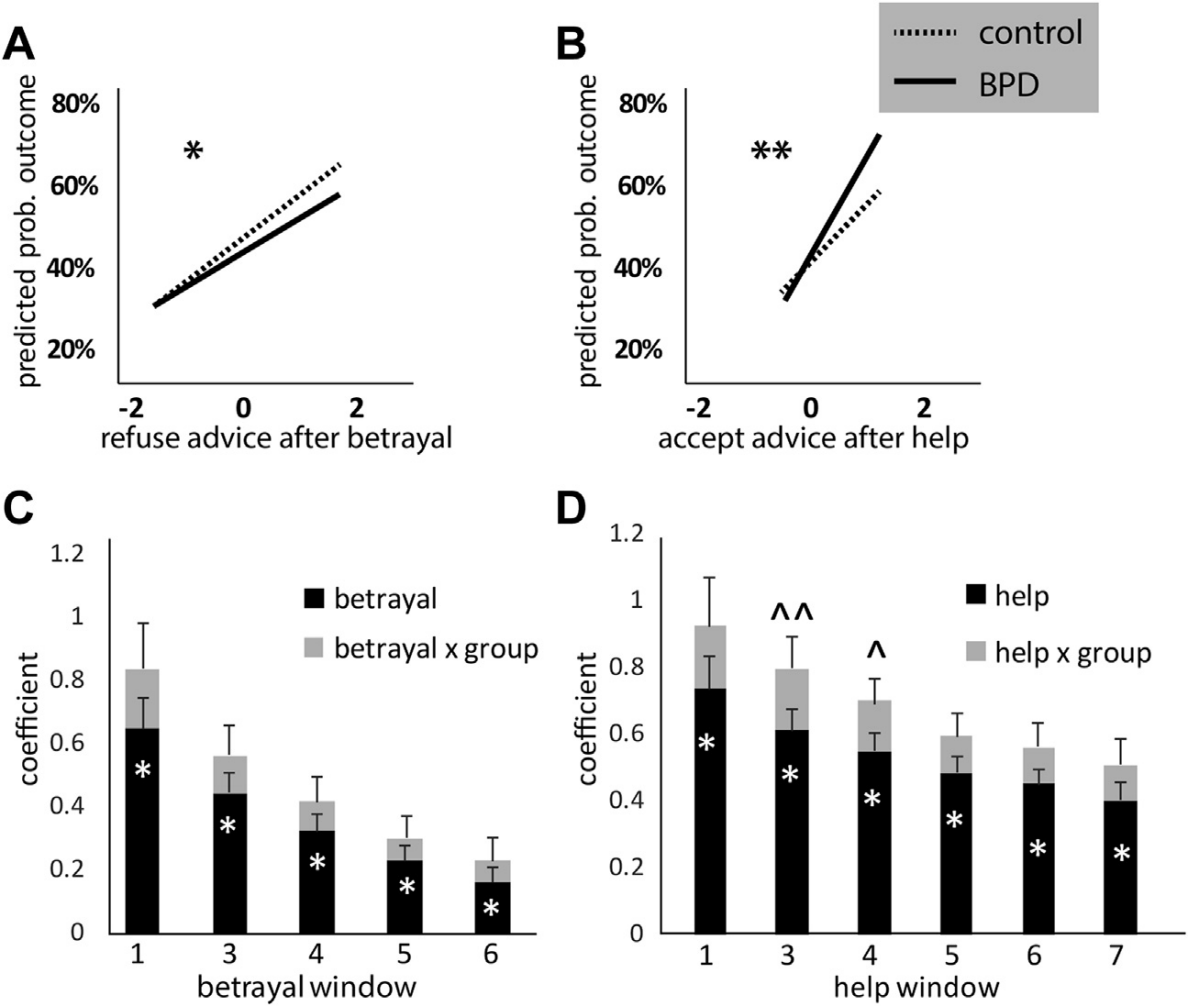
Both betrayal and help differently impact borderline personality disorder (BPD) and control subjects. The predicted probability of outcome differed in BPD vs. control subjects exposed to a recent instance of betrayal (incorrect advice) or help (correct advice). BPD subjects were more likely to refuse the advice after betrayal **(A)** (asterisk [*] indicates significant effect of group at *p* < .05) and take the advice after help **(B)** (double asterisk [**] indicates significant group × predictor effect at *p* < .05). **(C, D)** Results of a closer look at the use of betrayal and help over time. The bar graphs show regression results for a series of time windows, each with at least one betrayal **(C)** or help **(D)** event in the marked number of trials. For example, help window 1 means the advice was correct on the previous trial, help window 4 means the advice was correct on at least one of the previous 4 trials. All subjects showed diminishing use of these social cues with enlarging time windows; the significant group × predictor interaction in help windows 3 and 4 but not in the betrayal windows suggests a slower decrement of help use for decision making in the BPD group compared with the control group. This is not an effect that we observed for the use of betrayal. *Predictor *p* < .05, ^ˆˆ^predictor × group *p* < .05, ^ˆ^predictor × group *p* < .1.

To better understand the responses of BPD and control subjects to social cues in this interactive context, we next tested the predicted choices after recent betrayal (bad advice) or help (good advice) (Figure 4). We found a significant decrease in BPD patients in use of betrayal to avoid bad choices (BPD > control subjects, for betrayal within the last three trials, likelihood ratio χ^2^ statistic = −4.25, *p* = .039) (Figure 4A) and a trend toward a group × predictor interaction for increased use of help to find good choices in BPD patients (for help within the last three trials, BPD > control subjects, likelihood ratio χ^2^ statistic for group × predictor interaction = −3.79, *p* = .051).

We also tested the rate of decrement in weighting of recent help or betrayal. As expected, both groups used help or betrayal less as the window size expanded (Figure 4C, D), but both help and betrayal were significant predictors of outcome out to at least a seven-trial window. However, it was help (the positive social cue) not betrayal (the negative social cue) that showed a trend toward a group × predictor interaction (use of help decayed more slowly in the BPD than the control group).

### H4/5: Learning Rates Reveal Blunted Response to Increased Reward Volatility in People With BPD

**Figure 5:**
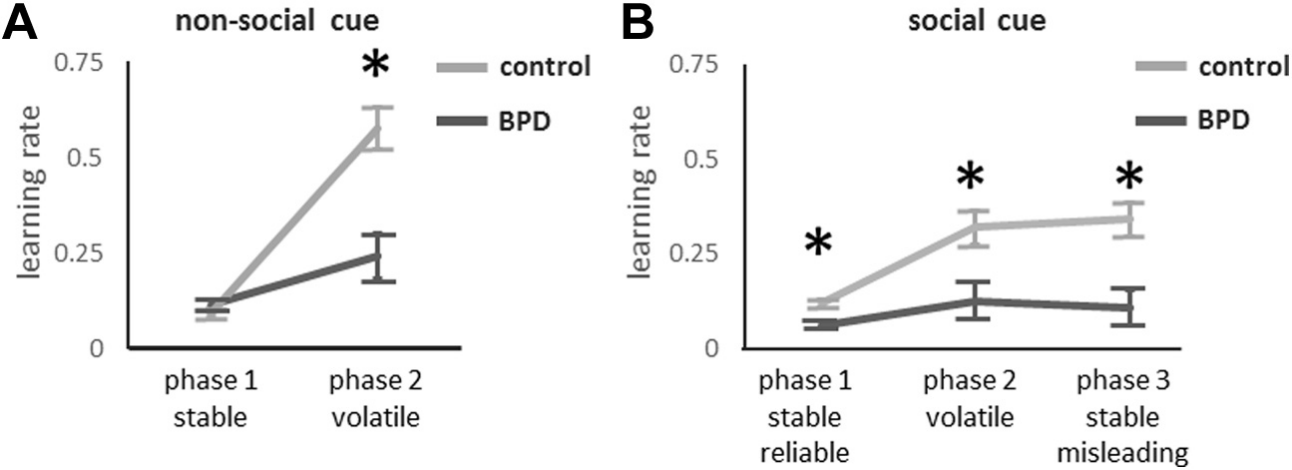
Learning rates were estimated for each individual, then differences were analyzed by group and condition. The asterisk (*) indicates significant between-group difference. **(A)** For learning from the nonsocial cue (color), there was a group × condition interaction (*F*_1,41_ = 19.78, *p* < .001). Learning was slow and not significantly different by group when reward probability was stable (*t* = −1.38, *p* > .05). However, when reward probability became volatile, the borderline personality disorder (BPD) group had a blunted response (less of an increase in learning) compared with control subjects (*t* = 4.12, *p* < .001). **(B)** For learning from the social cue, a group × condition interaction was also observed (*F*_2,40_ = 5.81, *p* < .01). Learning was slower in the BPD group than in the control group under all three conditions (stable reliable *t* = 4.02, *p* < .001, volatile *t* = 2.90, *p* < .01, and stable misleading *t* = 3.44, *p* < .005), and there was again a blunted response to volatility in BPD vs. control.

We modeled learning rates for nonsocial and social rewards during the stable and volatile phases of the task. We found that control and BPD subjects learned at similar low rates about nonsocial data during the initial phase when reward probability was stable (*t* = −1.38, *p* > .05) (Figure 5A). However, when reward probability became volatile (phase 2), control subjects increased their learning rate more than twice as much as did the BPD subjects (significant group × condition interaction: *F* = 19.78, *p* < .001) (Figure 5A). Learning from social cues was slower in BPD than in control subjects during all three phases of the task (stable reliable *t* = 4.02, *p* < .001, volatile *t* = 2.90, *p* < .01, stable misleading *t* = 3.44, *p* < .005), and response to volatility in BPD was significantly lower than in control subjects (group × condition interaction, *F* = 5.81, *p* < .01) (Figure 5B). These results were surprising: we had hypothesized faster learning rates in BPD in response to reward volatility; instead we observed a blunted response compared with control subjects for both nonsocial and social cues.

## Discussion

In this first study of the SVT in a patient population, we examined task performance in people with BPD, a condition defined by prominent interpersonal problems. In this extended interactive paradigm, women with BPD did indeed focus on social experience, weighting social over nonsocial cues to make decisions. However, we also found that a negative social experience (incorrect advice) was a less potent and less durable influence on subject choice than a positive social experience (correct advice).

Previous work in noninteractive paradigms, such as Reading the Mind in the Eyes and morphed face challenges, has identified a strong negative attribution bias [reviewed in 19, 20]. BPD patients attend quickly to negative faces and spend more time looking at them. A small number of studies have tested the response of BPD patients to social interaction games using brief paradigms. In the 10-round trust game, players with BPD responded with low initial trust and failure to coax defecting partners back to play (6). A key difference between the trust game paradigm and the SVT is that the latter combines social and nonsocial cues. The SVT allows direct investigation of the weighting of nonsocial versus social cues. For example, negative social experiences could impact the relative use of each cue type.

Unlike the reported problems in task performance with increased subclinical autism and psychopathy symptoms, our sample of women with BPD completed the SVT with final point scores similar to control subjects and used both social and nonsocial cues to make decisions. Nonsocial and social cues were weighted more heavily in the BPD group than in the control group. This may suggest that people with BPD are more attentive to the cues around them. Previous work describing learning in BPD has had mixed results. Work in brief nonsocial paradigms found that BPD state (emotional arousal) but not traits (Borderline Symptom List score) predicted problems with learning acquisition and vice versa for reversal learning (21). Others found no difference in reversal learning (22) but deficits in working memory in BPD (23).

In the extended and more complex social interaction in the SVT, we were able to examine not only low probability but also low reliability social rewards. In contrast to the defection (failure to coax) that was observed in the trust game after low payoff trials (6), we saw increased use of social cues under conditions of high social reward volatility here (in both groups, but BPD > control subjects), as if subjects were redoubling their efforts to remain socially engaged.

We extended previous reports of the effect of personality/mental health traits on SVT behavior by examining learning rates. We replicated the Behrens *et al.* (8) report that control subject learning rates increase under conditions of reward volatility. However, we were surprised to observe that BPD subjects showed only half the learning rate increase that control subjects did in response to nonsocial reward volatility, and barely responded at all to social reward volatility. One possibility is that BPD subjects assume higher baseline volatility of all environments and contingencies, such that high volatility is not surprising, and does not prompt updating. This is consistent with research demonstrating that early life adversity, especially neglect (i.e., volatility), is a key risk factor for BPD. Our observation that people with BPD decrease their self-referential language after confirmation of deception is consistent with this idea. The BPD subjects used fewer language markers that connote distress once they were informed of the deception, consistent with the idea that they harbor assumptions that the social world is unreliable. For someone sensitive to volatility, attending closely to cues but not updating rapidly may be adaptive. Future work could model prior beliefs about social and nonsocial cues to test our hypothesis that people with BPD assume higher baseline volatility.

This insensitive learning account of BPD social behavior is also consistent with a recent report describing a computational model of BPD trustee responses in the 10-round trust game (24). King-Casas *et al.* reported in 2008 that people with BPD fail to coax a defecting partner to re-engage in economic exchange (6). Their new paper describes a hierarchical belief model that significantly benefits from parameters describing a player’s own irritability and beliefs about the partner’s irritability. Here, irritability means the likelihood of retaliation on a low economic offer. BPD players were significantly less sensitive to their partners’ irritability than control players, and the authors suggest that this leads to missed opportunities to respond: a player does not coax if she does not detect early cues that the partner is becoming irritable or is likely to disengage.

Particular strengths of our approach here include the use of a patient population; in fact, we carefully screened participants for nonclinical status (control subjects) or significant symptoms (Diagnostic Interview for Borderlines - Revised score > 8 in the BPD group). Our subjects met and interacted with the confederate before starting the task, perhaps increasing their ability to engage with the task in a manner that reflects their real-world social behavior. The SVT is a lengthy interactive task that combines nonsocial (one’s own beliefs) with social (others’ counsel) for decision making at each trial. The task architecture includes orthogonal periods of volatility for the nonsocial and social cues, which allows us to model the relative use of each data source in decision making.

There are several limitations of this work. The sample is small and all female. We would expect gender differences in the expression of BPD and cannot generalize these results to men. We see our symptomatic patient group as a strength of this study, but our exclusion of people with fewer or less intense symptoms does preclude dimensional analysis of the impact of symptom burden on behavior. Also, we did not test what aspect of psychopathology (e.g., negative affect, anxiety, or negative attribution bias) is most correlated to the outcome measures. Future work will benefit from a larger sample with psychopathology control groups (depression, anxiety, and posttraumatic stress disorder) or a dimensional approach with subjects who vary in intensity of core symptoms, relevant comorbidities, and treatment history. Symptoms also fluctuate widely with time in BPD: future work should test the relationship of task behavior to subject emotional state by self-reports (e.g., Positive and Negative Affect Scale) or physiological arousal (e.g., galvanic skin response or pupillary dilation). From the opposite perspective, testing the impact of the task on subject state could further validate our finding of distress in subjects’ posttask language. To examine changes in task behavior over time, as we might wish to do to understand how subject learning changes with treatment in clinical practice, we will need interactive tasks that do not rely on deception and are more likely to work in repeated measures, as in an SVT-inspired task developed by Diaconescu *et al.* (11).

Future work should also examine social learning in BPD in settings that engage more realistic, and real-world, relationships. Schilbach *et al.* (25) have scored tasks based on whether the subject is interactive (vs. passive) and engaged (vs. dispassionate), with the truly interactive engaged task representing a more informative approach to social cognitive experiments, “second person neuroscience.” In this frame, emotional engagement is tightly linked to bodily experience and the complex and immediate dynamics of perceiving another’s action states. In our experiment, subjects do meet and briefly interact with their game partner (confederate) before the task. The SVT asks the subject to make decisions using the partner’s advice. The subject also knows that she and the game partner are incentivized to adjust their choices to the way the other behaves. Therefore, we see this task as interactive—the subject is playing the game in cooperation (competition?) with her game partner (or at least she thinks so). However, in terms of emotional engagement, the subject does not see or hear the game partner during the task, so no emotion is conveyed through bodily movements or language. Though some might argue that people with BPD are often surprisingly engaged based on little data, we think that this task likely does not meet the Schilbach *et al.* (25) criteria for an engaged task, and that future work could test this axis by including more possibility for engagement.

Our approach to modeling also has some limitations. We used mixed-effects regression models to compare our subjects’ behavior to an ideal Bayesian observer (a model without random effects). There is a potential tension here in terms of how much we account for individual variation in the processes that generate subjects’ behavior. As discussed above, we aim in the future to model additional parameters, such as subjects’ prior beliefs. In this initial effort to extend the work of Behrens *et al.* (8), we adhered closely to their approach. However, Diaconescu *et al.* 9, 11, 26 have used the Hierarchical Gaussian Filter, allowing individual differences and obtaining subject-specific estimates of approximate Bayesian inference. Modeling patient behavior using the hierarchical Gaussian filter may also detect subtler between-group differences (11).

Computational psychiatry, which focuses on the development of mechanistic models linking clinical symptoms to neurobiology and observed behavior through computational parameters, has already begun to describe the neurobiology of learning under volatile conditions (26) and to help mental health researchers improve prognostic prediction (27) and make plans to more precisely target therapeutics (28). A computational psychiatry of social interaction holds promise for honing existing treatments and building new ones in BPD and other disorders with prominent interpersonal symptomatology (29).
