# Supplementary material for: Differential Valuation and Learning From Social and Nonsocial Cues in Borderline Personality Disorder
Source: Biol Psychiatry. 2018 Dec 1;84(11):838–45. doi: 10.1016/j.biopsych.2018.05.020 (PMC6218635; doi:10.1016/j.biopsych.2018.05.020)
Supplement: Supplemental Material [file mmc1.pdf]

Differential Valuation and Learning From Social and Non-social Cues in Borderline Personality Disorder

Supplementary Information

Supplemental Methods and Materials

Social valuation task

On each of 290 trials (~45 minutes), subjects were presented with a binary choice between a green box and a blue box (non-social cue) **(Figure 1A)**. They also saw a point value associated with each color, and advice from a confederate about which color to pick (social cue). Before play, the subject met the confederate, interacted briefly, and together, subject and confederate were instructed in how to play the game. They were instructed to attend to three main concerns: how to choose a color, how to use the advice, and how points work. Specifically that: 1) one color would be more likely to be correct, and through feedback after each trial, the subject would be able to learn which color to choose, 2) that the confederate would give advice to the subject that may be helpful, or that may serve the confederate's own goals, and 3) that points are displayed on each of the two choices (green and blue) for each round, with correct choices rewarded by the displayed point value, and no possibility of losing points. Correct choices add the displayed point value to a total point tally. The tally was displayed as a growing bar at the bottom of each screen. If the bar grew long enough, the subject would win a $10 or $20 bonus. All subjects were in fact compensated $20 for the study visit and task completion. During a demonstration round, the subject observed the confederate choose to "give correct advice" and "give incorrect advice".

The task includes orthogonal variation in non-social (color) and social (advice) reward probability and reward volatility (frequency of changes in reward probability) (**Figure 1 B-D**). Behrens et al. found that healthy control subjects learn from variation in both non-social and social reward contingencies in the SVT (1).

Subject recruitment and screening

Initial screening during phone screen used the Diagnostic Interview for Personality Disorders BPD questions (2) and a series of brief questions to screen for other exclusion conditions. Based on phone screen results, potential subjects were invited to the lab, where we conducted an in-person semi-structured interview with a psychiatrist (SKF) including the Revised Diagnostic Interview for Borderlines (DIB-R (3)) and Structured Clinical Interview for DSM-IV (SCID (4)). To be included in the study, controls had no psychiatric conditions, and BPD subjects had no current substance dependence and no primary psychotic disorder. On the DIB-R, controls scored ≤ 4 (scaled total), BPD subjects scored ≥ 8 (scaled total). Included subjects also read English well (no history of special education, ≤ 11 errors on the Wide Range Achievement Test 4th Edition (WRAT-4) reading test (5)), had intact color vision, had no history of head injury or neurologic condition, and had no gait disturbance. See **Table 1**.

We also recorded subject education level, hours of work and/or schoolwork per week, current relationship status, and reading level (to detect differences among the more literate subjects in our sample, we used the more challenging North American Adult Reading Test (NAART) instead of the WRAT-4 score here) (6). See **Table 2**.

Self-report scales

The Borderline Symptom List (BSL-23) is a 23-item scale with established reliability (Cronbach's alpha 0.94-0.96) and validity (*r* = 0.96 versus the longer BSL-95, *r* = 0.87 versus the Beck Depression Inventory, and *r* = 0.48 versus the general psychopathology scale SCL-90) in initial psychometric studies (7). In our sample, 19 control subjects and 20 BPD subjects completed the scale, and Cronbach's alpha is 0.95.

The Beck Anxiety Inventory (BAI) is a 21-item scale is established reliability (Cronbach's alpha 0.94) and validity (*r* = 0.54 versus diary reports of anxiety) in an initial psychometric validation study (8, 9). In our sample, 20 control subjects and 20 BPD subjects completed the scale, and Cronbach's alpha is 0.95.

The Beck Depression Inventory (BDI-II) is a 21-item scale with established reliability (Cronbach's alpha 0.9) and validity (r = 0.71 – 0.86 versus a range of commonly used depression scales) in a large meta-analysis (10, 11). In our sample, 20 control subjects and 20 BPD subjects completed the scale, and Cronbach's alpha is 0.97.

The Structured Clinical Interview for DSM-IV Personality Questionnaire was not initially designed as a stand-alone instrument, but it has been found to have few false-negatives, and to be reliable compared to the clinician-administered version (12-14). In our sample, 20 subjects in each group completed this measure.

Regression models comparing cue weighting by group

Mixed effects regression models were used to test the impact of clinical group on behavior in response to each of our predictor variables. All models also included subject as a random variable (modelling both random intercept and random slope). Four models were compared for each variable: a full model including both clinical group and the interaction *v*-by-group as fixed effects (model #); a reduced model without the interaction effect (model p); a further-reduced model that included neither the interaction nor the main effect of clinical group (model n), and a fully-reduced model that contained only the random-effect for subject (model x). Likelihood ratio (LR) tests were used to compare nested models for model # compared to model p (to test for a significant interaction effect), for model p compared to model n (to test for a significant effect of clinical group), and for model n compared to model x (to test for a significant effect of predictor). The LR is a statistic that is a more positive number as model fit improves.

$$\textit{likelihood ratio chi-square statistic} = -2*(LR_{biggermodel} - LR_{smallermodel})$$

Therefore, in a case when the bigger model (the one with more terms) improves on the smaller model, the likelihood ratio chi-square statistic will be a negative number. To understand the directionality of the

impact of specific model terms on outcome, we examined the estimated coefficient for each term. For the group term, control was coded as 0 and BPD as 1, so positive coefficients can be interpreted to mean that BPD makes the outcome more likely.

In order to test for apparent statistical differences that may be explained by our having treated each binary decision as independent in the statistical model, we verified the results with a permutation procedure where group membership is randomized and test statistic recalculated. We found statistically significant ($p < 0.05$) values for likelihood ratios (chi-square) tests comparing the permuted to the reported models.

Learning model

The authors summarize the model as follows: “The 2-armed bandit model in hBayesDM updates expected value according to:

$$V(t+1) = V(t) + A * (R(t) - V(t))$$

Where V is the expected value on trial t, A is the learning rate, and R is the outcome of the chosen option on trial t. V is only updated for the chosen option. The values (V) are entered into a softmax function with an inverse temperature (Tau) that measures how sensitive subjects are to the differences in expected value between choice options. Higher and lower values for Tau indicate responding more deterministically and randomly with respect to the learned expected values, respectively.” (personal communication with Nathaniel Haines)

The actual implementation is available at https://github.com/CCS-Lab/hBayesDM/blob/master/exec/bandit2arm_delta.stan.

Supplemental References

1. Behrens TE, Hunt LT, Woolrich MW, Rushworth MF (2008): Associative learning of social value. *Nature*. 456:245-249.
2. Zanarini MC, Frankenburg F.R., Sickel, A.E., Yong, L. (1996): The Diagnostic Interview for DSM-IV Personality Disorders. . In: Mclean Hospital LftSoAD, editor. Belmont, Mass.
3. Zanarini MC, Gunderson, J.G., Frankenburg F.R., Chauncey, D.L. (1989): The Revised Diagnostic Interview for Borderlines: Discriminating BPD from other axis II disorders. *Journal of personality disorders*. 3:10-18.
4. First MB, Spitzer, R.L., Gibbon, M., Williams, J.B.W. (2007): Structured Clinical Interview for DSM-IV-TR Axis I Disorders-Patient Edition. In: Institute BRDNYSP, editor. New York, New York.
5. Wilkinson GS, Robertson, GJ. (2006): Wide Range Achievement Test 4. Lutz, FL: Psychological Assessment Resources.
6. Uttl B (2002): North American Adult Reading Test: age norms, reliability, and validity. *J Clin Exp Neuropsychol*. 24:1123-1137.
7. Bohus M, Kleindienst N, Limberger MF, Stieglitz RD, Domsalla M, Chapman AL, et al. (2009): The short version of the Borderline Symptom List (BSL-23): development and initial data on psychometric properties. *Psychopathology*. 42:32-39.
8. Fydrich T, Dowdall D, Chambless DL (1992): Reliability and Validity of the Beck Anxiety Inventory. *Journal of Anxiety Disorders*. 6:55-61.
9. Beck AT, Epstein N, Brown G, Steer RA (1988): An inventory for measuring clinical anxiety: psychometric properties. *Journal of consulting and clinical psychology*. 56:893-897.
10. Wang YP, Gorenstein C (2013): Psychometric properties of the Beck Depression Inventory-II: a comprehensive review. *Revista brasileira de psiquiatria*. 35:416-431.
11. Steer RA, Ball R, Ranieri WF, Beck AT (1999): Dimensions of the Beck Depression Inventory-II in clinically depressed outpatients. *J Clin Psychol*. 55:117-128.
12. Ball SA, Rounsaville BJ, Tennen H, Kranzler HR (2001): Reliability of personality disorder symptoms and personality traits in substance-dependent inpatients. *Journal of abnormal psychology*. 110:341-352.
13. Ekselius L, Lindstrom E, von Knorring L, Bodlund O, Kullgren G (1994): SCID II interviews and the SCID Screen questionnaire as diagnostic tools for personality disorders in DSM-III-R. *Acta psychiatrica Scandinavica*. 90:120-123.
14. Jacobsberg L, Perry, S., Frances, A. (1995): Diagnostic agreement between the SCID-II screening questionnaire and the Personality Disorder Examination. *J Pers Assess*. 65:428-433.
